# “It's more than just a conversation about the heart”: exploring barriers, enablers, and opportunities for improving the delivery and uptake of cardiac neurodevelopmental follow-up care

**DOI:** 10.3389/fped.2024.1364190

**Published:** 2024-05-24

**Authors:** Bridget Abell, David Rodwell, Karen J. Eagleson, William Parsonage, Ben Auld, Samudragupta Bora, Nadine A. Kasparian, Robert Justo, Steven M. McPhail

**Affiliations:** ^1^ Australian Centre for Health Services Innovation and Centre for Healthcare Transformation, School of Public Health and Social Work, Faculty of Health, Queensland University of Technology, Brisbane, QLD, Australia; ^2^ Centre for Accident Research & Road Safety—Queensland (CARRS-Q), School of Psychology and Counselling, Faculty of Health, Queensland University of Technology, Brisbane, QLD, Australia; ^3^ Queensland Paediatric Cardiac Service, Queensland Children’s Hospital, South Brisbane, QLD, Australia; ^4^ Faculty of Medicine, The University of Queensland, Brisbane, QLD, Australia; ^5^ Health Services Research Center, University Hospitals Research & Education Institute and Department of Pediatrics, University Hospitals Rainbow Babies & Children’s Hospital, Case Western Reserve University School of Medicine, Cleveland, OH, United States; ^6^ Heart and Mind Wellbeing Center, Heart Institute and the Division of Behavioral Medicine and Clinical Psychology, Cincinnati Children’s Hospital Medical Center and the Department of Pediatrics, University of Cincinnati College of Medicine, Cincinnati, OH, United States

**Keywords:** congenital heart disease, neurodevelopmental follow-up, implementation, health services research, barriers and enablers

## Abstract

**Introduction:**

Surveillance, screening, and evaluation for neurodevelopmental delays is a pivotal component of post-surgical care for children with congenital heart disease (CHD). However, challenges exist in implementing such neurodevelopmental follow-up care in international practice. This study aimed to characterise key barriers, enablers, and opportunities for implementing and delivering outpatient cardiac neurodevelopmental follow-up care in Australia.

**Methods:**

an exploratory descriptive qualitative study was conducted with healthcare professionals across Australia who had lived experience of designing, implementing, or delivering neurodevelopmental care for children with CHD. Online semi-structured interviews were conducted using a guide informed by the Consolidated Framework for Implementation Research to explore contextual influences. Interview transcripts were analysed using a rapid qualitative approach including templated summaries and hybrid deductive-inductive matrix analysis.

**Results:**

fifty-two participants were interviewed. Perceived barriers and enablers were organised into six higher-order themes: factors in the broader environmental, economic, and political context; healthcare system factors; organisational-level factors; provider factors; patient and family factors; and care model factors. The largest number of barriers occurred at the healthcare system level (service accessibility, fragmentation, funding, workforce), while service providers demonstrated the most enabling factors (interprofessional relationships, skilled teams, personal characteristics). Strategies to improve practice included building partnerships; generating evidence; increasing funding; adapting for family-centred care; and integrating systems and data.

**Discussion:**

Australia shares many similar barriers and enablers to cardiac neurodevelopmental care with other international contexts. However, due to unique geographical and health-system factors, care models and implementation strategies will require adaption to the local context to improve service provision.

## 1 Introduction

Some of the most prevalent ongoing concerns for children living with congenital heart disease (CHD) are developmental, behavioural, and psychological difficulties. This includes an elevated risk for cognitive, motor, language, and socioemotional delays (1–3) as well as challenges with attention and executive functioning (4, 5). While these may often be mild to moderate deficits, they can have considerable impacts on a child's education, social participation, health service use, functional independence, mental health, and quality of life (6–8). Consequently, early identification of neurodevelopmental delays or disorders is a pivotal component of care for children with CHD at risk for this sequalae (9, 10). This includes a combined approach of longitudinal surveillance of all high-risk children; neurodevelopmental screening to assess concerns; standardised, formal, performance-based evaluation to identify developmental disorders; and timely referral for intervention (9).

Despite the recognized importance of neurodevelopmental follow-up care in this population, challenges exist in current practice. Surveys from the United States (11), Canada (12), Europe (13), and South Africa (14) report highly variable and potentially suboptimal practices while also identifying systemic barriers to implementation and uptake of care. Commonly, challenges are associated with a lack of skilled staff or resources, families needing to travel distances for care, limited provider knowledge and awareness, and financial concerns (including insurance coverage, out of pocket costs, provider reimbursement, and service delivery costs) (15). While published guidance from the American Heart Association (9) outlining the neurodevelopmental follow-up needs of children with CHD could be perceived as an enabler, challenges have been noted in applying these recommendations outside their original United States context (12, 13, 16).

To advance the uptake of neurodevelopmental follow-up care for children with CHD internationally, the role and influence of barriers and enablers across different settings, countries, or contexts must be considered (17). Targeted strategies can then be used to address these identified barriers and enablers. Tailoring service delivery to context may also improve care practice and outcomes (18, 19). Thus, comprehensive assessment of local and national contexts to identify influential determinants is a critical first step for improving implementation and delivery of cardiac neurodevelopmental care within health systems.

As an example, Australia presents a particularly contrasting context to that of the United States, where much of the research examining implementation of neurodevelopmental follow-up for children with CHD has been conducted. Australia employs a government-funded universal healthcare system that prioritises equitable, low-cost access for all, coupled with a nationally funded disability support scheme^1^, optional private health insurance system, and greater reliance on community-based primary care^2^ (20). The country also has unique geographical and cultural characteristics, including a low population density outside urban centres, making centralisation of care challenging. With the recent publication of the National Strategic Action Plan for Childhood Heart Disease in Australia (21), which identifies delivery of neurodevelopmental follow-up as a core part of CHD care, understanding service delivery in this specific national context is a key priority. However, to our knowledge, no studies have systematically examined factors influencing delivery and uptake of neurodevelopmental care for children with CHD in Australia.

The purpose of this study was to characterise key barriers and enablers to implementing and delivering outpatient neurodevelopmental follow-up care for children with CHD in Australia. We used a descriptive qualitative approach to understand the current national context of service delivery, highlight clinical and service gaps, and identify targets for implementation strategies or new pathways to improve delivery and access to care. In doing so we aimed to generate evidence to support ongoing efforts to expand and adapt neurodevelopmental follow-up care beyond the North American context.

## 2 Materials and methods

### 2.1 Design

We undertook a qualitative, interview-based context assessment exploring stakeholders' perceptions of designing, implementing, or delivering neurodevelopmental follow-up care for children with CHD in Australia. We used this term to encompass services providing neurodevelopmental surveillance, screening, evaluation and/or therapy, unless otherwise stated. An exploratory descriptive qualitative design (22) allowed for in-depth exploration of real-world experiences, including barriers and enablers to providing effective care for this population. The study was informed by the Consolidated Framework for Implementation Research (CFIR) (23) as a means of grounding the context assessment within theoretical constructs known to influence implementation and effectiveness. These are grouped across five major domains, each representing a different aspect of implementation: Intervention Characteristics, Outer Setting, Inner Setting, Characteristics of Individuals, and Process.

Approval was granted by the Children's Health Queensland Hospital and Health Service Human Research Ethics Committee (LNR/21/QCHQ/73748) prior to study commencement. Reporting of recruitment, data collection and analysis was guided by the COnsolidated criteria for REporting Qualitative Research (24).

### 2.2 Sample and recruitment

Healthcare professionals from across Australia who had lived experience of designing, implementing, or delivering neurodevelopmental follow-up care for children with CHD were recruited. We sought a range of perspectives, from those who were routinely involved in cardiac neurodevelopmental care, to others who provided developmental paediatric or cardiac care more broadly. We used a combination of purposive and snowball sampling to include participants across a range of geographical locations, clinical settings, clinical disciplines, leadership levels, and years of experience. Clinical members of the research team identified potential participants through existing professional and clinical networks in Queensland. Similarly, potential participants in other regions of Australia were identified by contacting Australia-wide study partners.

An initial email about the study was sent to potential participants in each Australian state and territory by the local study partner. Those who expressed interest were then contacted by the project coordinator via email to provide participant information and schedule an interview. All participants were informed that participation was voluntary and would not impact their employment or professional relationship with study partners. Informed consent was obtained prior to starting the interview. A single reminder email was sent by the project coordinator if there was no response within two weeks of initial contact. Where included participants suggested more potential interviewees for the study, the same procedures were followed. Our final sample size was determined by a combination of thematic saturation and data sufficiency across key participant demographics (25).

### 2.3 Data collection

Online, semi-structured interviews were conducted from August 2022 to February 2023. Interviews were scheduled based on each participant's preferred timing and platform (Zoom, Microsoft Teams, phone). Most interviews were one-on-one; however, several participants chose group interviews (one interview had three participants, two interviews had two participants). Two experienced PhD qualified health service researchers (BA, female and DR, male) conducted the interviews. BA has previous clinical experience as an exercise physiologist in the Australian healthcare sector. Interviews lasted between 30 and 60 min. Audio recordings of the online interviews were professionally transcribed. Transcripts allowed revisiting of data to maintain the participant's original voice, increasing validity of the analysis. Phone interviews were not recorded due to logistical challenges. While detailed notes were taken for phone interviews, verbatim quotes were not recorded for these participants. Participants' professions, locations and other demographic characteristics were collected.

A semi-structured interview guide (Supplementary Data Sheet 1) was developed by BA and checked for face and content validity by the clinical team members. The guide comprised several open-ended questions, divided into two sections: (1) barriers and enablers to neurodevelopmental follow-up of children with CHD in Australia; and (2) current gaps and “blue sky” ideas to improve care in the future. In Section 1, prompts were aligned with the CFIR to capture barriers and enablers across a range of potential domains and constructs. Findings from both sections were highly interrelated and together form the context assessment.

### 2.4 Data analysis

The transcripts were analysed using a rapid qualitative approach including structured templates and hybrid deductive-inductive analysis of matrix displays^3^ (26). This approach aligned with our exploratory descriptive design as well as a need for pragmatic methods to synthesise a large amount of data in a limited timeframe to feedback to partners and inform ongoing, dependent study activities. Table 1 outlines the six stages of rapid analysis and describes how we conducted each stage within this study.

**Table 1:** Six stages of rapid qualitative analysis and methods used to conduct each stage.

Rapid analysis stage	Methods applied
1. Creating a summary template	The summary template (Supplementary Table 1) was developed in Microsoft Word and aligned with the sections and questions of the interview guide. Section 1 was divided into the CFIR domains of inner setting, outer setting, and individuals. Factors captured in these domains were to be classified as barriers (–) or enablers (+) to care. A column for capturing quotes was also included
2. Test driving the template with a transcript	Two researchers (BA and DB) independently summarised the same two transcripts using the template
3. Amending the template and retesting	BA and DB met to compare and discuss summaries. There was strong agreement between the summaries. After clarification of some definitions, the template was found to be sufficient for the remaining analysis
4. Dividing the data and making summaries of the remaining data	The remaining transcripts were split evenly between BA and DR for analysis. Each researcher independently summarised their allocated transcripts and spot-checked the other's summaries
5. Creating a matrix of summarised content	Using Microsoft Excel, data from all summaries were consolidated into several matrices to visualise findings and assist with synthesis into themes and selection of exemplar quotes. Three separate worksheets were created: one each for findings related to perceived barriers, enablers, and opportunities. Matrices were organised with individual participants on the horizontal axis (grouped by State or Territory) and relevant findings from each summary on the vertical axis (one idea/point per cell, grouped by CFIR domain, where appropriate). See Supplementary Table 2 for an example matrix
6. Synthesizing data into themes	Codes/lower-order themes were identified in each worksheet using an inductive approach to examine the content for similarities, differences, and interconnections both across the interviews (i.e., the horizontal axes) and across factors (i.e., the vertical axis). Higher-order themes were then synthesised inductively. We also used CFIR to guide deductive analysis of codes into higher order themes where appropriate. However, as developmental follow-up care for CHD sits across multiple settings and systems, we often experienced challenges determining if factors should be mapped to inner or outer domains. In these cases, we mapped across both domains if they contained relevant constructs (e.g., “Fragmentation of care” was mapped to “Partnerships & Connections” in Outer Setting and “Communications” in Inner Setting)

We also conducted a form of synthesised member checking to enhance data credibility and validity (27). Synthesised results and themes were presented to a selection of participants (*n* = 9) in a series of online meetings to confirm resonance with their own experience and offer opportunity for further input.

## 3 Results

Study partners identified and invited 123 stakeholders to participate. A total of 52 participants took part in 48 interviews: 43 conducted using Zoom or Microsoft Teams, and five phone interviews. Participant characteristics are provided in Table 2. We interviewed healthcare professionals from a range of seniority levels and clinical disciplines including nursing, paediatrics, cardiology, and allied health. Participants were predominantly female, with the majority working in metropolitan hospital-based settings. At least one participant was recruited from every Australian State and Territory, except for the Australian Capital Territory.

**Table 2:** Characteristics of the 52 healthcare professionals interviewed.

Demographic characteristic	Frequency (%)
Gender	
Female	39 (75)
Male	13 (25)
Clinical discipline	
Neonatologist	8 (16)
General paediatrician	7 (13)
Clinical nurse consultant	6 (12)
Developmental paediatrician	5 (10)
Occupational therapist	5 (10)
Physiotherapist	5 (10)
Speech pathologist	5 (10)
Paediatric cardiologist	4 (8)
Psychologist	3 (6)
Social worker	2 (4)
General practitioner	1 (2)
Neuropsychologist	1 (2)
Organisational role	
Healthcare provider	26 (50)
Senior healthcare provider (program, team, or clinical lead)	19 (37)
Executive or medical director	7 (13)
Service setting	
Hospital	41 (81)
Community	11 (19)
Service location	
Metropolitan	42 (81)
Regional	10 (19)
State or territory	
Queensland	15 (29)
New South Wales	10 (19)
Northern territory	9 (17)
Victoria	6 (12)
Western Australia	6 (12)
Tasmania	4 (8)
South Australia	2 (4)

**Figure 1:**
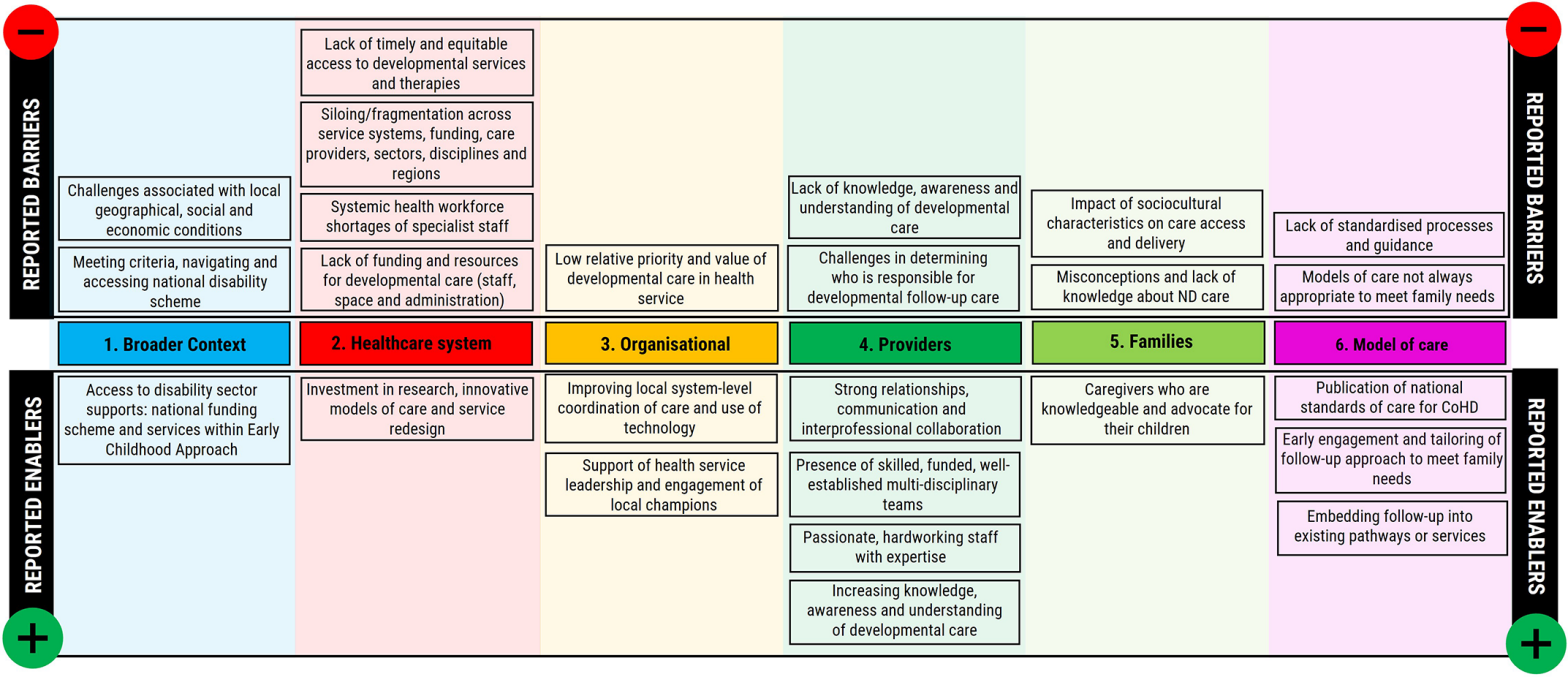
Perceived barriers and enablers to implementing and delivering neurodevelopmental care for children with CHD in Australia, grouped by colour-coded higher-order theme.

The perceived barriers and enablers identified in the transcripts were organised into six higher-order themes: broader environmental, economic, and political context; healthcare system factors; organisational-level factors; provider factors; patient and family factors; and care model factors (Figure 1). Identified barriers and enablers were also mapped across all CFIR domains and 27 individual constructs, suggesting comprehensiveness of results (Supplementary Table 3).

Most themes contained both barriers, which hindered the implementation and delivery of follow-up care, and enablers which supported service delivery. The largest number of barriers occurred at the healthcare system level, while service providers demonstrated the most enabling factors. Analysis suggested that barriers were not always mutually exclusive as several had points of intersection. For example, the barriers of service accessibility, lack of funding, and workforce shortages were interrelated. These three barriers were also the most frequently reported concerns across all participants.

### 3.1 Theme 1: broader context

#### 3.1.1 Environmental and socioeconomic challenges

The most frequently reported environmental challenge to the delivery of neurodevelopmental services in Australia was geographical. Specifically, the large dispersion of families across the country, often distant from tertiary care settings. This requires families to travel long distances to access specialised care and makes equitable staffing and delivery of follow-up services challenging. Additionally, participants reported that limited health infrastructure and internet connectivity in regional areas created further inequities in service access and availability for these families.

Context specific socioeconomic challenges were also noted in some regions of the country. Prioritisation and delivery of neurodevelopmental follow-up care for children was impacted by complex social determinants of health such as poverty, transportation, food insecurity, weather, domestic violence, access to childcare, care giver substance misuse, and foster care. As one participant described: “*I can't get near them with medical problems. I got to go—I've got to get all the social problems out first, you know?*”*.* (*P26, Paediatrician, Northern Territory*) These environmental and social challenges were most acutely experienced in the Northern Territory. Participants described a local context in which families faced difficulties travelling to access neurodevelopmental follow-up care due to seasonal weather events (monsoonal rains), a lack of wrap around supports (family-friendly accommodation and transport), or culturally insensitive policies (travel, family escorts).

#### 3.1.2 Disability sector and national disability insurance scheme

Australia's disability sector and government funded national disability insurance scheme were acknowledged by participants as having a small but significant role in supporting neurodevelopmental follow-up care for children with CHD. For children with qualifying co-morbidities or diagnosed developmental disorders the insurance scheme enabled access to lower-cost, locally based intervention services. However, children with CHD do not automatically qualify for support and challenges persist for parents with misguided expectations of the scheme, understanding and meeting eligibility criteria (including age limits and functional diagnoses), and navigating and accessing approved services. Improving connections between the health and disability sector to increase knowledge about disability support services within and beyond this scheme (e.g., free Early Childhood Approach playgroups) was perceived as a critical enabler of more appropriately integrating this complementary sector into predominantly health-based neurodevelopmental follow-up pathways.

### 3.2 Theme 2: healthcare system factors

#### 3.2.1 Accessibility and availability of developmental services and therapies in Australia

Participants from all disciplines and regions of Australia (*n* = 40) described the most significant challenge to be access for families to healthcare services to support neurodevelopmental follow-up care in a timely and equitable manner. The key concern was growing demand within a public (government-funded) health system already at capacity and experiencing significant workforce and funding constraints. Consequently, long waiting lists (up to 12–24 months) for both evaluation and intervention were frequently reported by participants from hospital-based and community services. However, as a large proportion of neurodevelopmental follow-up care for children with CHD is done in community, these services were strained. Timely access to developmental paediatricians, allied health providers, psychologists, and child development services were all flagged as challenges by participants. The scope of this problem across multiple regions and healthcare disciplines is highlighted in Table 3.

**Table 3:** Quotes from participants supporting the widespread challenge of developmental health service access in Australia.

Limited access to required specialists for assessment		
“*If there are any developmental assessments needed by a developmental paediatrician, it's really challenging to find anybody who will see them. A general paediatric clinic isn't really set up for that. And so, I think that's by far the biggest barrier to having paediatric assessments done*” *[P35]*	Cardiologist	Western Australia
“*Child Development's in a crisis state. There are families under immense stress because they're unable to understand and manage their child with neurodevelopmental difficulties. And we are failing them as a public service because they're waiting more than a year to see us*” *[P25]*	Child Development Unit Lead	Queensland
“*We have a lot of problems accessing decent psych, well not decent, but any sort of psych services*” *[P40]*	Program Manager	Western Australia
Long waiting lists nationwide		
“*I've never known the waiting list to be less than six months. If you were on a six-month list, you were doing well*” *[P14]*	Psychologist	New South Wales
“*A lot of them, they are waiting a significant amount of time on our waiting lists, so a lot of those issues are going unaddressed. So, we really worry about that clinical risk of children sitting on our waiting list and potentially deteriorating*” *[P27]*	Child Development Unit Lead	South Australia
“*The wait list here at [Hospital] alone, we have 1,000 kids currently waiting outside of recommended timeframes for developmental health follow up, developmental care.*” *[P17]*	Hospital Executive	Queensland
“*I spend a lot of time telling parents, get yourself on a wait list …like there's not enough clinicians and then the clinicians that are here don't have enough time*” *[P22]*	Paediatrician	Northern Territory

While access to services was a common challenge nationally, participants noted greater disparities for particular groups of children. Firstly, First Nations communities were perceived to have inequitable access to universal screening, and maternal and child health services, leading to delayed presentation. Secondly, those living in regional or rural areas experienced greater access challenges with a limited number of health services often available, high turnover of clinicians and variable access to specialists.

“*If you look at the models of child development services in those regional areas, they do exist, but they're quite limited and then they don't have any of those subspecialties available to them*”*. [P37, Child Development, South Australia]*

Affordability of neurodevelopmental evaluation and intervention was highlighted as a lesser challenge. This included both the time and money required for families to attend public sector appointments, as well as the high cost of choosing to access care though private providers. The cost of private services was described to be “*hundreds and hundreds, if not upwards of a thousand dollars*” [P14, Psychologist, New South Wales]. While paying to see private providers presented a good option for some families, this did not guarantee timely access to care:

“*When it comes down to private psychologists or private paeds [paediatricians], private occupational therapists, with them, yet again, it's the same issue [waiting lists]. So even though our parents would be willing to spend the money to access private health services, they would still have to undergo the huge wait times.*”*[P41, Neonatologist, Western Australia]*

#### 3.2.2 Fragmentation and siloing within and across the system

A similarly strong theme identified across interviews (*n* = 32) was the challenge of delivering holistic neurodevelopmental follow-up care in a system which was experienced as being uncoordinated and fragmented. Silos of care were reported within and beyond the health system, driven by a lack of communication, collaboration, data sharing, integration and coordination between different service systems, geographical regions, care providers, clinical disciplines, and sectors (see Table 4 for examples). Given that neurodevelopmental follow-up care for children with CHD sits in a unique space at the intersection of multiple services, disciplines and government sectors, this siloing becomes a major challenge for care delivery and funding.

**Table 4:** Quotes from participants highlighting multiple challenges with fragmentation and siloing of neurodevelopmental follow-up care in Australia.

Challenge	Exemplar Quote	Interview participant and discipline
Fragmented funding system	“*The different layers of funding, so having state government funding and federal government funding for different services. You know, NDIS [disability] is Federal, hospitals are State. GPs [primary care] are Federal, community services often locally funded from local councils. I think that fragmented funding system is a big problem*”	P11, Neonatologist
Fragmented health service system	“*I think moving between multiple services is a challenge… It's sort of like walking into a whole other country where there's different language and different rules and so I think that can be really challenging for parents*”	P18, Clinical Nurse Consultant
Lack of cross-sector integration	“*If we think about those four big areas where kids and families receive care, health, education, disability, social services, it's really hard to get them to talk with each other, really hard to get them to shift beyond,* ‘*This is my patch, this is what I need in order for you to access services from me.*’ *There's that barrier around getting different departments of government to talk and cooperate and focus on good outcomes for children*”	P38, Paediatric Disability Leader
Lack of data or care integration within hospitals	“*We don't have any standardised linkages between kids who are cared for in cardiology, cardiac surgery and the developmental clinics*”	P8, Developmental Paediatrician
Poor handover and communication between hospitals and health services	“*Sometimes the communication is less than ideal. We sometimes have children who've had major cardiac surgery who get discharged back into a regional or rural area and we don't get told about them*”	P44, Developmental Paediatrician (regional)
Poor communication between clinical providers	“*The families kind of bounce back and forth between a paediatrician and a cardiologist and neither speaking to each other*”	P20, Psychologist

For example, several participants highlighted the disconnect between the health, education, and disability sectors, including separate funding and service delivery models. Similarly, care was not always perceived to be shared well when transitioning from hospital to community services, and gaps were also noted in the transition from paediatric to adult services. Difficulties navigating complex care systems were perceived as a challenge for both families and healthcare providers. This was compounded by the fact that many providers interviewed were not sure about how developmental care was supported in other states or regions of the country, outside their own. This presented a challenge in supporting families who needed to access care across state borders or in a region different to where their surgical care was provided.

#### 3.2.3 Funding and resources

Concerns were raised about the ability of current funding allocation to adequately resource the delivery and sustainment of neurodevelopmental follow-up services across Australia. Participants (*n* = 27) highlighted the key challenge of meeting the growing demand for these services while facing constrained funding for staff, training, physical space and infrastructure, early intervention services, and multidisciplinary care models [see Supplementary Table 4, A1 for more supporting quotes].

“S*o, for me we do not have enough resources. Definitely, we do not have enough public resources in Queensland to deliver to the demand.*” *[P25, Child Development Unit Lead, Queensland]*

Participants expressed frustration with a perceived misalignment of state and federal funding priorities with the delivery of follow-up care. Namely, that the allocation of health service funding is often skewed towards acute care provided by tertiary centres, which is at odds with the need for long-term, local delivery of neurodevelopmental follow-up care. As a result, community paediatric and allied health services were perceived as considerably underfunded, contributing to staff shortages and long waiting lists [Supplementary Table 4, A2].

“*We spend millions on operations. And then a few thousands that really could make a difference to their life, and society's life, is just hard to get.*” *[P15, Occupational Therapist, New South Wales]*

Other important challenges include regional inequities in funding and resource allocation, limited resourcing of community supports and non-governmental organizations, the ways service eligibility criteria were influenced by funding, and an unsustainable reliance on philanthropic funding for some service delivery [Supplementary Table 4, A3].

Several participants provided examples where financial support and investment had been a key enabler in improving neurodevelopmental follow-up care. Funding by individual health services to support dedicated roles, program implementation and coordination, innovative models of care, developmental training, and the redesign and extension of services was reported as a key enabler. The federal government's growing research investment in the sector was also recognized as a positive drive towards change.

#### 3.2.4 Workforce and staffing

Participants in every state and territory (*n* = 27) reported significant barriers related to systemic health workforce shortages, particularly of those providers with the required expertise to deliver neurodevelopmental follow-up to children with CHD. This was most acutely felt in publicly funded community services, with shortages of developmental paediatricians, allied health professionals and nurses common. This challenge was compounded by high staff turnover due to ongoing difficulties in recruiting and retaining staff. Private sector competition, inadequate service funding, limited training opportunities in neurodevelopmental assessment, and variable interest in the field were suggested as potential contributing factors [Supplementary Table 4, B1].

“*The availability of experienced, qualified staff to be able to provide that follow-up care [is a challenge]. There just seems to be shortages everywhere you look of—you know, we find it really hard to recruit here.*” *[P37, Child Development Unit Lead, South Australia]*

Additionally, there was perceived to be a lack of funding for dedicated roles to support neurodevelopmental follow-up of children with CHD, including nurse coordinators, and administrative assistants. Ultimately, these workforce shortages were reported to have direct impacts on clinic capacity, scope of services provided, and the ability to triage and see families in a timely manner.

### 3.3 Theme 3: organisational level factors

#### 3.3.1 Health services do not often prioritise or recognise the value of long-term neurodevelopmental care for children with CHD

Many participants (*n* = 19) expressed frustrations that despite evidence of its importance, neurodevelopmental care is still not given sufficient priority within local healthcare systems. It was suggested that its long-term value for children is not well understood by hospital executives and funders. Consequently, the lack of leadership buy-in to invest in developmental paediatrics and early childhood services hampers the allocation of resources needed to deliver neurodevelopmental follow-up care for children, including those with CHD [Supplementary Table 4, C1].

* *“*I just don't see that commitment [from hospital] to really understanding the value of this work.*” *[P20, Psychologist, Victoria]*

While cardiologists were generally perceived as supportive of neurodevelopmental follow-up care, participants felt that medical outcomes were still prioritised over long-term development.

“*I don't think it's really high priority. And really, most cardiac services are just preoccupied with making children survive and getting better. And, that's actually not a criticism, it's—it's just the reality of the resourcing.*” *[P13, Paediatric Cardiologist, New South Wales]*

Participants also raised challenges they had experienced when advocating for follow-up programs in a health system focused on acute, hospital-based care which often viewed neurodevelopmental follow-up care for children with CHD as an “optional extra” [Supplementary Table 4, C2–3].

“*At the end of the day, our health system prioritises acute care, prioritises adult care, and it prioritises hospital-based over community care. And that's just the way it is.*” *[P23, Developmental Paediatrician, Queensland]*

“*I think psychology and neurodevelopmental care is still seen as the icing on the cake as opposed to an absolutely essential, routine, ordinary, necessary part of cardiac care.*” *[P14, Psychologist, New South Wales]*

Conversely, the presence of influential individuals or leaders who were committed to supporting and championing neurodevelopmental follow-up, and fostering collaboration among colleagues, was reported to be a key enabler of success. The importance of such engagement across both hospital-based and community settings was highlighted by participants [Supplementary Table 4, C4].

“*But I also think we've got really, really strong leadership. And leadership who are really present, and really respectful of everybody's role and advocate enormously for that. So, I think creating change is much easier when you've got the support of strong leaders.*” *[P15, Occupational Therapist, New South Wales]*

#### 3.3.2 Improving hospital systems and coordination

Participants (*n* = 17) also described how improving local system-level care coordination had positively impacted on their ability to provide neurodevelopmental follow-up. Commonalities included utilizing and integrating technology, implementing efficient systems, coordinating appointments, maintaining continuity of care, and triaging referrals. Streamlining processes, optimizing communication, and minimizing barriers for families better enabled access to comprehensive neurodevelopmental follow-up care. For example, some hospitals reported using care coordinators to gather information and liaise with families to prevent children from falling through the cracks [Supplementary Table 4, D1]. Others used flags on the electronic medical record to trigger a neurodevelopmental assessment for children with CHD at routine paediatric or cardiology follow-up clinics:

“*The fact that it's built into electronic records and automated means that hopefully people won't fall through the gaps.*” *[P36, Cardiologist, Victoria]*

A small number of participants highlighted a lack of standardised measures and data systems for assessing the outcomes and effectiveness of neurodevelopmental follow-up care. This made capturing data about their own services challenging and impacted their ability to benchmark across services and perform quality improvement activities.

### 3.4 Theme 4: provider factors

#### 3.4.1 Relationships, networks and interprofessional collaboration

Participants (*n* = 20) described the strong enabling role that positive relationships, effective communication and interprofessional collaboration played in supporting care delivery. At a service provision level this included collaborative relationships between different hospital-based teams as well as with community providers such as allied health clinicians, general practitioners, Aboriginal health services, and child health nurses. In this way, service provision could be complementary and care better transitioned and integrated for families [Supplementary Table 4, E1].

“*[It helps] if we engage community services within our program and work together, because the community services interact with the patients and their families a lot more frequently than we do.*” *[P33, Neonatologist & Paediatrician, Northern Territory]*

Several participants also highlighted the supportive role that school-based psychologists and guidance counsellors played in their care pathways. Finally, it was perceived that the paediatric development and heart disease communities in Australia are collegial and collaborative, citing buy-in from state-wide clinical networks as a clear enabler.

“*I think probably a big part of the success is the congenital heart disease community is a pretty tight knit community and everybody partners together really well.*” *[P3, Clinical Nurse Consultant, Queensland]*

#### 3.4.2 Skilled and established multidisciplinary teams

Despite overarching workforce issues, many health providers (*n* = 18) did report the existence of funded, well-established, and skilled multidisciplinary teams in their services. The presence of these collaborative teams was a key enabler in providing neurodevelopmental follow-up to children with CHD.

“*We have low staff turnover, so I guess it gives you that ability to kind of build a stable and consistent pathway of care.*” *[P1, Allied Health Team Leader, Queensland]*

In these teams, each member brought expertise within their respective role, and success was usually attributed to good communication and building trust with each other and families. The provision of local capacity building opportunities in neurodevelopmental assessment/evaluation was highlighted as an important strategy to foster the creation and sustainment of skilled teams. One participant described the outcome of such training in a regional service:

“*We've noticed over the past three years, certainly the clinicians that have stayed around and most actually have, is that they've really built up those observational assessment skills and some of those trans-disciplinary skills.*” *[P24, Social Worker, Queensland]*

#### 3.4.3 Passionate and generous health care providers

Skilled, hardworking, and generous staff who are passionate about caring for children and improving practice are a positive attribute of current neurodevelopmental follow-up care. Staff members often went above and beyond their regular duties to enhance their knowledge and skills for the benefit of patients. Across the disciplines of cardiology, paediatrics, nursing, and allied health, participants described staff members who invested significant personal time and effort to build and improve developmental screening and assessment services [Supplementary Table 4, F1].

“*One of my colleagues has spent the last five years building this developmental follow up clinic, like there was absolutely nothing before. And she has put in hours and hours of her free time, including working in it for free for the last two or three years to try and make it happen.*” *[P22, Neonatologist & Paediatrician, Northern Territory]*

#### 3.4.4 Knowledge and understanding of neurodevelopmental care

Participants highlighted the enabling role played by recent increases in recognition from cardiologists and paediatricians about the need for ongoing neurodevelopmental follow-up for children with CHD, and benefits of targeted and appropriate interventions.

“*Now the focus is actually on preparing families for developmental issues so that they're more aware, so they will actually recognise problems or advocate for the child, or seek help as well because it's more than just a conversation about the heart.*” *[P36, Cardiologist, Victoria]*

Comments by other participants however, suggested that development was still not always top of mind for many clinicians, dampening the strength of this enabler [Supplementary Table 4, G1].

“*I'll be honest, I don't routinely think about making a neurodevelopmental referral for the bulk of patients that I see in clinic who've had surgery.*” *[P35, Paediatric Cardiologist, Western Australia]*

Those interviewed also felt many providers still had insufficient knowledge and understanding about when and how to identify neurodevelopmental concerns associated with CHD and provide appropriate referral options. In practice this often resulted in poor quality referrals and gaps in clinical communication.

#### 3.4.5 Blurred lines of responsibility

A recurring theme was the complexity associated with determining clinical responsibility for neurodevelopmental follow-up, with tensions between different public sectors, healthcare providers, and parents. Many attributed this to the complex interplay between acute hospital-based cardiac care, followed by long-term community-based developmental follow-up care, as well as a lack of guidance about the roles and expectations of each health provider group or sector.

“*I think the biggest challenge is working out who owns the problem. And certainly, our hospital systems don't see it as our responsibility, they see developmental problems as community services’ responsibility. But there's no over-arching coordination of that, so it's really fragmented.*” *[P11, Neonatologist, Victoria]*

Several participants described cardiologists “buck passing” their responsibility to support neurodevelopmental follow-up for these children [Supplementary Table 4, H1]. Others felt clinicians may be hesitant to discuss mental health concerns in particular because that could “*open a Pandora's box and let it all out… and if there's no clear pathway to follow [for management] a lot of clinicians will just prefer not to open the box at all*” [P13, Paediatric Cardiologist, New South Wales]. As a result, participants believed families were often heavily burdened with advocating for their child's neurodevelopmental follow-up care and navigating the complex service landscape on their own [Supplementary Table 4, H2].

“*…developmental delay doesn't tell you what your child support needs are. So, you need to have the skills, knowledge, and confidence, to find and advocate for the right supports for your child.*” *[P38, Paediatric Disability Lead, Queensland]*

### 3.5 Theme 5: family factors

#### 3.5.1 Knowledge and understanding of neurodevelopmental care

While growing recognition about the importance of neurodevelopmental follow-up for children with CHD within the medical community was acknowledged as an enabler, many felt that the broader Australian community still did not acknowledge the association between CHD and neurodevelopmental diagnoses [Supplementary Table 4, I1]. Among parents of children with CHD, gaps in understanding persist regarding the importance of neurodevelopmental follow-up care, and their capacity to access services and resources varies. Some parents were also perceived to have misconceptions or variable understanding about the role of different services available to support them, which hindered their uptake or could lead to unrealistic expectations and disappointment. These issues were compounded by a perceived lack of support from the health sector to help families navigate the system and advocate for services [Supplementary Table 4, I2]. On the other hand, when parents were provided with the opportunity to advocate knowledgably for their child, access to neurodevelopmental follow-up care was enabled [Supplementary Table 4, I2].

#### 3.5.2 Sociocultural characteristics of children and families

Engagement of culturally and linguistically diverse (CALD), refugee, and First Nations communities was a commonly reported challenge (*n* = 21 participants). For many of these families, limited English language and low health literacy contributed to difficulties accessing and understanding healthcare services. Cultural differences were also reported to play a role.

“*I think that culturally for Aboriginal families, there's a very broad tolerance of difference. And so, I don't think they identify disability in the same way that some non-Aboriginal families do.*” *[P42, Paediatrician, Western Australia]*

Those interviewed had also observed competing priorities between health, community/cultural events and socioeconomic pressures in these communities which affected attendance at follow-up appointments.

“*There's life happening for them in the community. And this [follow-up] is less important and it's important but it's on the hierarchy of importance. What's important in their lives at that time? It's not that important.*” *[P33, Neonatologist & Paediatrician, Northern Territory]*

Finally, carer educational level, mental health status, and previous positive or negative experiences with the hospital system were also considered important determinants of access to neurodevelopmental follow-up care. For example, parents who had experienced traumatic stress in relation to their child's cardiac diagnosis or hospitalisation may be more reluctant to return to the hospital setting for follow-up care.

“*These families have been—they've been dreading bad outcomes from the time they had their morphology scan. They have lived through trauma before their baby is born. And now we are here telling them to come back to hospital for what?*” *[P33, Neonatologist & Paediatrician, Northern Territory]*

### 3.6 Theme 6: model of care factors

#### 3.6.1 Lack of standardised processes and guidance for neurodevelopmental follow-up care

Participants across many regions (*n* = 19) pointed out the lack of structured programs, processes, and evidence-based guidance for delivering neurodevelopmental follow-up care to children with CHD in Australia [Supplementary Table 4, J1].

“*I don't think we have a framework about exactly what type of patient should have a routine assessment. If they all should, or only certain risk factors, or cohorts? There's not really a lot of, I think, structure to that.*” *[P35, Paediatric Cardiologist, Western Australia]*

“*You know, we're really lacking things like a very—a formal model of care, practice framework, clinical guidelines.*” *[P37, Child Development Unit Lead, South Australia]*

This, combined with the silos across services already described, was perceived to contribute to the high variability in how neurodevelopmental follow-up was delivered to children with CHD across the country. Some providers expressed concern that this contributed to inequitable experiences of care for families. Additionally, without systematic processes for identifying and capturing children with CHD who may be at risk, ongoing surveillance is challenging. As a result, the onus for driving follow-up is placed on individual clinicians and families, with children more likely to fall through the gaps [Supplementary Table 4, J2).

“*It is reliant on cardiologists, and importantly cardiac nurses, identifying the need for that additional input rather than having a structured program that just kicks in automatically and supports those kids and families. So, I think it's not an ideal model.*” *[P39, Paediatric Cardiologist, South Australia]*

The impending publication of National Standards of Care for Childhood-Onset Heart Disease was however, identified as a potential enabler for standardising future clinical practice.

#### 3.6.2 Leveraging existing programs and pathways to improve access

Many participants expressed concerns about the viability of follow-up programs, or clinics, exclusively for children with CHD in Australia. Rather, embedding their follow-up into other existing funded community or hospital services, including maternal and child health, child development, feeding clinics, Indigenous liaison services, private allied health providers, and paediatrics clinics was suggested as a potential enabler to increase access to care. Embedding formal, standardised and widely-available screening tools, such as the Ages & Stages Questionnaire (ASQ), into already existing personal health records was perceived as a positive strategy in several health services. Incorporating multidisciplinary team members into cardiology outreach clinics was also seen as a successful strategy for neurodevelopmental assessment in regional areas. Additionally, well-established and structured follow-up programs for all at-risk neonates (e.g., pre-term, low birth weight) were supporting a proportion of children with CHD who also met eligibility criteria. Expansion of funding and eligibility criteria was suggested as a way for these programs to support more children with CHD in the future. Finally, several participants raised the suggestion of a broader systems-based approach to neurodevelopmental follow-up, expanding beyond these specific populations [Supplementary Table 4, K1].

“*So, I guess they're [children with CHD] a group within a broader group, and in some ways that broader group are at risk and need a similar service. So, at a systems level, when you're thinking of how we should follow up our young people with significant congenital heart disease, it would be, I think, in a broader context of children with serious medical comorbidity.*” *[P23, Developmental Paediatrician, Queensland]*

#### 3.6.3 Designing models of care to meet family and provider needs

Many participants (*n* = 24) commented on specific characteristics of the models of care used in Australia within the context of neurodevelopmental follow-up which either supported or hindered service delivery. Most neurodevelopmental follow-up services and pathways in Australia do not extend beyond young childhood, which was identified as a key shortcoming of current care. In particular, the ability to engage with and surveil school-aged children, who had “aged-out” of child health or developmental services was a challenge. Additionally, a lack of culturally responsive models of care for First Nations people was perceived to be a barrier in delivering care to this population. However, some participants also highlighted instances where services had integrated high levels of cultural support through provider training, adapted assessment tools, and Indigenous staff members, which in turn enabled more equitable care.

“*It's just understanding that we have to find a middle ground between the Western way and the traditional Aboriginal way. And to find a way to work together.*” *[P30, Paediatric Nurse, Northern Territory]*

Early engagement and neurodevelopmental support of families in hospital was reported to be a key positive component of current care pathways. After this, being able to provide a flexible and tailored approach to follow-up based on context and family needs was a key enabler. This included the use of virtual care for connecting families with health providers, and health providers with each other, where appropriate.

“*Telehealth has been working really nicely. That people can communicate with team members better but also just kind of access care for young, busy families that are struggling to keep up with everything.*” *[P20, Psychologist, Victoria]*

Finally, clinicians felt that the use of simple global screening tools, such as the ASQ (rather than batteries of tests) was helpful.

“*Having tools that everyone can use is amazing, so having the ASQ and ASQ-track is awesome. And some more tools like that, and it's just easy. You just apply it. It's not completely easy. But it's just that standardised tool, standardised screening stuff is just so helpful.*” *[P22, Paediatrician, Northern Territory]*

### 3.7 Opportunities to improve delivery and sustainment of services

**Figure 2:**
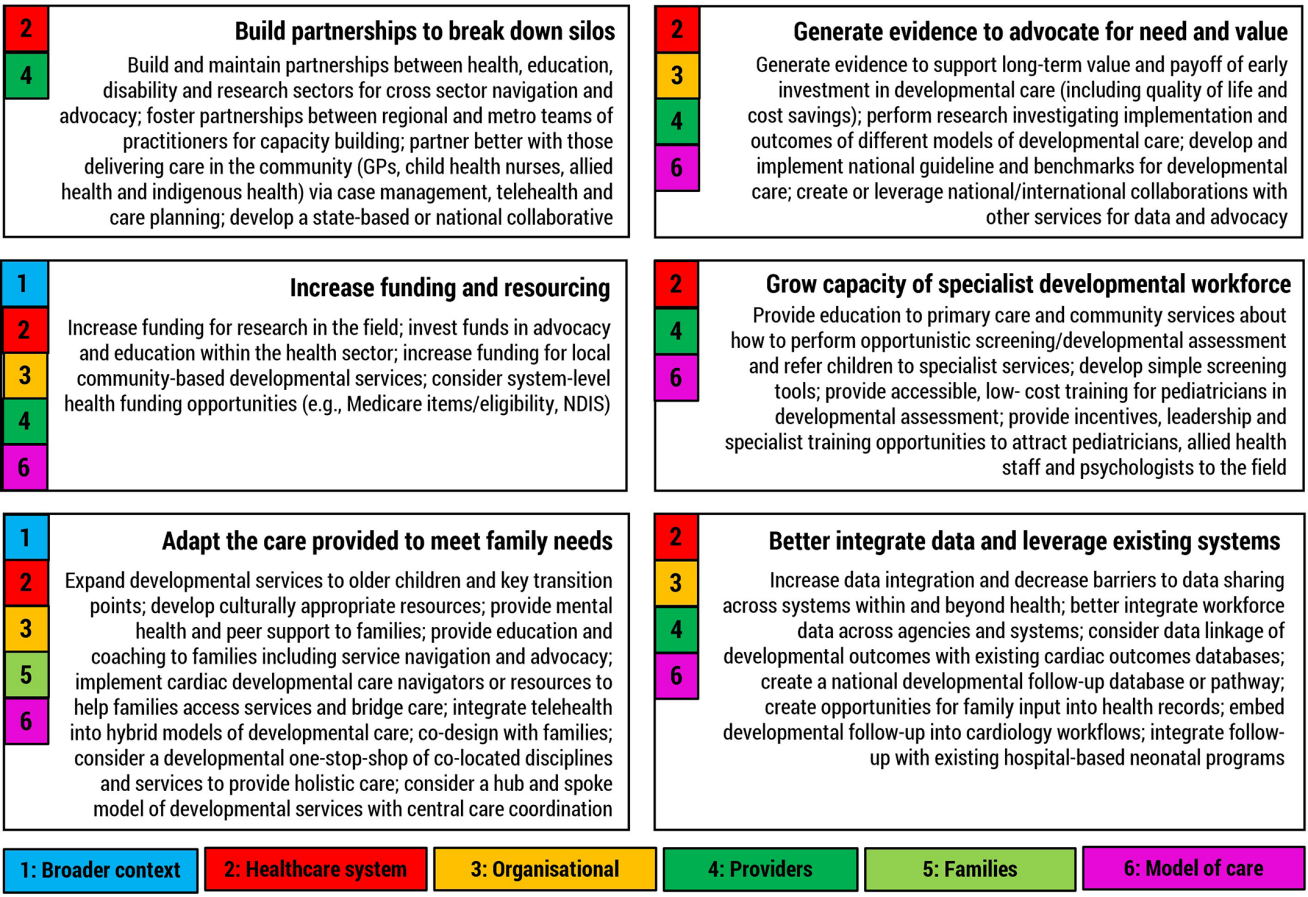
Suggested strategies for improving neurodevelopmental follow-up of children with CHD in Australia. Numbers represent higher order barrier or enabler theme the category maps to.

When asked about opportunities to improve neurodevelopmental follow-up for children with CHD in Australia, participants made suggestions which either addressed identified barriers or capitalised on enablers. Figure 2 highlights how these suggestions were grouped into six broad categories, each of which was mapped to factors at multiple levels of service implementation. For example, building partnerships both addressed barriers at the healthcare system level and capitalised on enablers at the provider level. Quotes to support each of these groups of strategies are provided in Supplementary Table 5.

## 4 Discussion

This qualitative study provides a unique insight into factors at multiple levels which influence the implementation and uptake of neurodevelopmental follow-up care for children with CHD in Australia. The wide range of factors identified reflects the complexity of delivering such care across the implementation contexts studied. Despite geographic and organizational differences in the characteristics of participants, barriers and enablers were generally similar across the country. Our findings also provide insight into strategies that could be adopted to directly target known barriers and enablers to improve neurodevelopmental follow-up care more systematically in the future.

### 4.1 Comparison with international literature

Our findings suggest that, for the most part, Australia does not present a wholly unique context for cardiac neurodevelopmental follow-up care. Participants in our study highlighted many barriers which are consistent with those reported in other countries such as lack of a skilled, specialist workforce (11, 12), limited funding and resources (11, 12, 28–30), variable health provider knowledge and awareness (14, 31), burdening of families (14, 28, 32, 33), living distant from care (34, 35), family socioeconomic status (34), lack of systematic process and pathways (12) and a disconnect across different healthcare settings (11, 12). In particular, the challenge of adequately funding neurodevelopmental follow-up, both at a program/service and system level was shared internationally. As in Australia, this was perceived to drive the barriers related to workforce (including lack of psychologists and social workers) and service capacity (28). However, the limitations in care access imposed by insurance coverage, service reimbursement and payor contracts observed in several US-based studies (11, 32, 34) were not significant concerns in the Australian context. Australia's public health insurance system may, to some extent, overcome the access difficulties for uninsured children.

On the contrary, limited availability of developmental services for assessment and intervention was one of the most significant challenges observed in our study, however this issue is rarely mentioned in studies of US-based cardiac neurodevelopmental follow-up care. One explanation could be that the focus of most cardiac neurodevelopmental follow-up care research in the US to date has been limited to specialised follow-up programs based at large urban multispecialty care centers (15). While these programs are commonly available for developmental evaluation of high-risk children with CHD (11), such research may understate the challenges experienced in delivering and accessing developmental services more broadly across the country and particularly for those children outside the reach of urban centers, or experiencing socioeconomic disadvantage that limits their participation. For example, the comparative absence of these cohort specific follow-up programs in Australia, along with greater dispersion of families from tertiary hospitals, places greater burden on general community-based developmental services to perform these roles. Despite offering care closer to home, providing neurodevelopmental follow-up via this generalist model creates clear challenges when these services are already overburdened (36) or lack specialist staff. Indeed, studies from South Africa, Canada, and the United Kingdom, three other healthcare systems with few structured programs, have also highlighted challenges for children with CHD accessing publicly funded developmental services and long waitlists across the care spectrum from screening to intervention (14, 30, 33). Even US-based cardiac neurodevelopmental follow-up programs have reported issues with availability of evaluation in the schooling years (37).

These findings would suggest that establishing more specialised hospital-based programs for children with CHD could be an important strategy to improve access to neurodevelopmental follow-up in Australia. Such clinics also capitalise on other identified enablers such as promoting cardiac neurodevelopmental care, supporting research and quality improvement, providing care from a highly skilled multidisciplinary team, and integration with cardiology reviews. However, Australia's unique contextual challenges of geography, sociocultural characteristics of families, and publicly funded services make it difficult to replicate the organisation and structure of US-based programs in an equitable, cost-efficient, and accessible way. These challenges, as well as the resource intensive nature of high-risk neurodevelopmental follow-up and early intervention more broadly in the Australian context have previously been described (38, 39). Consequently, as suggested in research from Canada and Europe (12, 13), there is a need to adapt and test models of care more appropriate for the local context. It is most likely that cardiac neurodevelopmental follow-up care in Australia requires some combination of structured hospital-based programs and well-supported, systematically applied community-based pathways. This is reiterated in the upcoming Australian National Standards of Care for Childhood-onset Heart Disease (40) which do not mandate hospital-based follow-up programs but rather a systematic and coordinated model of care delivered via partnerships across the health system. Further research is required to understand how this type of care could be implemented successfully in practice.

### 4.2 Enablers for service model design and delivery

Historically, studies have focused mostly on the barriers, rather than enablers to practice change. In this study, however, there was a specific focus on the enabling factors underpinning successful implementation across all parts of the delivery system. In doing so we have highlighted key considerations for design and delivery of care to capitalise on identified enablers. These mostly comprise opportunities to act across levels of the system which may be more amenable to change (service design, families, providers, organisations) compared to broader system-level challenges.

Firstly, this study has highlighted the important role that health providers, families and organisational leaders play within the system delivering cardiac neurodevelopmental follow-up care. This is consistent with other research describing leadership buy-in, provider awareness, dedicated and skilled teams, empowered caregivers, and interprofessional partnerships as key factors in follow-up program success (11, 29, 30, 41). Consequently, any efforts to improve service delivery should first include capitalising on these key human resources. Indeed, half of the key improvement strategies suggested by study participants (capacity building, partnerships, and meeting family needs) adopted this approach. Because of the similarity in challenges across the country, there are opportunities to build national clinical and research networks to conduct multisite projects and share learnings. This will also help to address some of the siloing observed in the system and assist with care navigation for service providers. The impending publication of the Australian National Standards of Care will be a key resource for this strategy as it calls for national and international collaboration. Greater collaboration with existing international networks such as the Cardiac Neurodevelopmental Outcome Collaborative may also be beneficial.

Another key opportunity at the service level involves capitalising on enablers related to models of care. For example, research in North America has highlighted the importance of two of these suggested enablers: hospital-led care coordination (42) and leveraging existing resources and data systems in complimentary services (29). In particular, service-led care coordination will be key for reducing the burden experienced by families and health professionals in navigating the complex service environment and will be essential if implementing shared hospital and community-based follow-up pathways. Additionally, the importance of having flexibility in follow-up care to meet family needs and preferences cannot be overlooked, but how this can be balanced with greater national standardisation requires further examination, both in Australia and the international literature. Finally, consideration of the role of children with CHD in the paediatric developmental healthcare system as a whole will be important when considering the best way to leverage existing resources and services.

### 4.3 Time for a system based approach?

Cardiac neurodevelopmental follow-up care is complex and involves interactions between families, health care providers, clinical services, sectors, and funders at all levels of the system. Additionally, the array of barriers and enablers identified in this study suggests that a comprehensive approach to improving the provision of cardiac neurodevelopmental follow-up care is required which targets determinants across multiple contextual levels. This contrasts with existing attempts to improve neurodevelopmental follow-up care provision which focus largely on single issues, such as program improvement (43, 44) or family or provider knowledge (43, 45). This study also highlights a complex interplay between several key barriers in the healthcare system including funding, access, workforce shortages and recognition of the value of neurodevelopmental follow-up care. Understanding the inter-relationship between these barriers and their relevance to the broader health care landscape is also important when considering the best targets for interventions to improve cardiac neurodevelopmental service delivery. Namely, it is pertinent to consider if addressing one key barrier may in turn reduce others, or support change in other parts of the system.

As an example, evidence suggests that the perception of value and relative importance of an intervention is one of the strongest predictors of implementation success (46, 47). Yet, participants in this study expressed a sentiment that the Australian health sector has a bias to fund acute medical care such as cardiac surgery and does not value appropriately investing in the long-term sequalae of CHD. This view was also highlighted in a Canadian study where providers felt their cardiac neurodevelopment services did not have the same profile or support in their organisations as their acute-care counterparts (28). Consequently, increasing the value proposition for cardiac neurodevelopmental follow-up services at an organisational and national level via evidence generation, guidelines, benchmarking, advocacy, education, and partnerships (Figure 2) should be a key strategy. However, our relational analysis of contextual barriers in this study suggests this may also be a key leveraging strategy, with such a change likely to have flow on effects to improve individual service funding, the number and type of services available, and encourage greater workforce participation in the field. Such an approach could be adopted at the organisational level to begin with, however, to be most effective, system-wide solutions involving collaboration between different stakeholders, and policy-level changes and infrastructure improvements (e.g., requirements for staffing and service delivery as currently outlined in the National Standards of Care), will be required. This system-wide approach would have a considerable impact on barriers at the national healthcare system level and may in turn improve family and service outcomes (48).

Adopting a systems-based approach to improving cardiac neurodevelopmental care in Australia is also likely to have positive spillover effects for other cohorts of children (49). As highlighted by study participants and evident in the literature, many of the higher-level implementation challenges are shared with similar high-risk paediatric cohorts and by families trying to access developmental care more generally. For example, geographical challenges, long waitlists, difficulties in system navigation, siloed care, limitations in workforce capacity, and service availability have all been reported as barriers to accessing children's health, development and disability services by First Nations, culturally and linguistic diverse, and regional populations in Australia (50–52) and other international contexts (53). While there is little published literature about the challenges of pre-term follow-up in Australia, international evidence supports shared system-level barriers across the pathway from surveillance to early intervention (54, 55). Understanding how the CHD community could use this multiplicity of shared determinations to work collaboratively with the broader neonatal and paediatric developmental communities to drive system-level change should be a key focus of ongoing work. This would be likely to benefit all groups and have application beyond the Australian context.

### 4.4 Strengths and limitations

One of the key strengths of this study is its qualitative nature, allowing an in-depth exploration of implementation context directly from the perspective of stakeholders. Moreover, our sample was large and diverse, representing perspectives about cardiac neurodevelopmental follow-up care from across the country. Additionally, the use of an implementation science framework allowed systematic identification of issues across multiple levels of the system. This contrasts with much previous knowledge about barriers and enablers to cardiac neurodevelopmental follow-up care which comes from retrospective evaluations of single centre programs or quantitative surveys of care providers. Finally, the attainment of data saturation, and use of transcripts, verbatim quotes, clinician team members, and member checking strengthened the rigour of our findings.

Study limitations relate mostly to the participant sample. Despite repeated recruitment attempts, primary care physicians and cardiologists were most likely underrepresented in the sample. Consequently, their experiences of cardiac neurodevelopmental follow-up care may not have been fully captured within the study findings. Additionally, participants from Queensland were overrepresented in the sample, which may have placed more emphasis on issues in this region. However, analysis suggests this was not the case with reported themes being consistent across all states and territories. The perspective of families was not sought for this context assessment and should be followed up with further research. Finally, stakeholders self-selected to participate in the interviews which could mean a limitation of perspectives to those most engaged in the field. Conversely, it could also be argued that these particular participants hold the most informed views and are therefore critical to understanding context.

### 4.5 Summary

The results suggest that a comprehensive approach to improving the provision of neurodevelopmental follow-up is required which tackles the array of identified barriers and enablers across multiple layers of the system. Adopting a system approach is likely to have positive ripple effects for the paediatric developmental healthcare system but will require enabling policies and prioritisation of investment in primary healthcare and developmental service capacity.

## Data Availability

Data are not publicly available due to information that could compromise the privacy of research participants if published. However, excerpts of the transcripts relevant to the study that support the findings are available on request from the corresponding author.

## Footnotes

^1^Hewett R. National Disability Insurance Scheme: A Quick Guide Canberra: Commonwealth of Australia (2022) [cited 2023 15/9/2023]. Available online at: https://www.aph.gov.au/About_Parliament/Parliamentary_departments/Parliamentary_Library/pubs/rp/rp2122/Quick_Guides/NationalDisabilityInsuranceScheme.

^2^Australian Institute of Health and Welfare. Health System Overview Canberra: Australian Institute of Health and Welfare (2022) [cited 2023 27/8/2023]. Available online at: https://www.aihw.gov.au/reports/australias-health/health-system-overview.

^3^Hamilton A. Qualitative Methods in Rapid Turn-around Health Services Research. Powerpoint Present 2013 Va Hsrd Cyberseminar Spotlight Womens Health. [Powerpoint ]. Online(2013) [cited 2023 August 10 2023]. Available from: https://www.hsrd.research.va.gov/for_researchers/cyber_seminars/archives/780-notes.pdf.

## References

[1] Latal B. Neurodevelopmental outcomes of the child with congenital heart disease. Clin Perinatol. (2016) 43(1):173–85. 10.1016/j.clp.2015.11.012 26876129

[2] Huisenga D La Bastide-Van Gemert S Van Bergen A Sweeney J Hadders-Algra M. Developmental outcomes after early surgery for complex congenital heart disease: a systematic review and meta-analysis. Dev Med Child Neurol. (2021) 63(1):29–46. 10.1111/dmcn.14512 32149404 PMC7754445

[3] Loblein HJ Vukmirovich PW Donofrio MT Sanz JH. Prevalence of neurodevelopmental disorders in a clinically referred sample of children with CHD. Cardiol Young. (2023) 33(4):619–26. 10.1017/S1047951122001469 36094009

[4] Jackson WM Davis N Calderon J Lee JJ Feirsen N Bellinger DC Executive functions in children with heart disease: a systematic review and meta-analysis. Cardiol Young. (2021) 31(12):1914–22. 10.1017/S1047951121001074 33766182

[5] Tarek Hasan M Shaban Abdelgalil M Elbadawy MA Mahmoud Elrosasy A Elkhadragy A Awad KA. Prevalence of attention deficit/hyperactivity disorder (ADHD) in congenital heart diseases (CHD), a systematic review and meta-analysis. Eur Heart J. (2022) 43(Supplement_2):2522. 10.1093/eurheartj/ehac544.2522

[6] Gonzalez VJ Kimbro RT Cutitta KE Shabosky JC Bilal MF Penny DJ Mental health disorders in children with congenital heart disease. Pediatrics. (2021) 147(2):e20201693. 10.1542/peds.2020-1693 33397689 PMC7849200

[7] Ladak LA Hasan BS Gullick J Gallagher R. Health-related quality of life in congenital heart disease surgery in children and young adults: a systematic review and meta-analysis. Arch Dis Child. (2019) 104(4):340–7. 10.1136/archdischild-2017-313653 29572215

[8] Marelli A Miller SP Marino BS Jefferson AL Newburger JW. Brain in congenital heart disease across the lifespan: the cumulative burden of injury. Circulation. (2016) 133(20):1951–62. 10.1161/CIRCULATIONAHA.115.019881 27185022 PMC5519142

[9] Marino BS Lipkin PH Newburger JW Peacock G Gerdes M Gaynor JW Neurodevelopmental outcomes in children with congenital heart disease: evaluation and management: a scientific statement from the American Heart Association. Circulation. (2012) 126(9):1143–72. 10.1161/CIR.0b013e318265ee8a 22851541

[10] Ware J Butcher JL Latal B Sadhwani A Rollins CK Brosig Soto CL Neurodevelopmental evaluation strategies for children with congenital heart disease aged birth through 5 years: recommendations from the cardiac neurodevelopmental outcome collaborative. Cardiol Young. (2020) 30(11):1609–22. 10.1017/S1047951120003534 33143781

[11] Basile NL Brown Kirschman KJ Dempster NR. Psychosocial, neurodevelopmental, and transition of care practices provided to children with CHD across North American cardiac clinics. Cardiol Young. (2023) 33(2):235–41. 10.1017/S1047951122000488 35184773

[12] Bolduc ME Rennick JE Gagnon I Majnemer A Brossard-Racine M. Canadian developmental follow-up practices in children with congenital heart defects: a national environmental scan. CJC Pediatr Congenit Heart Dis. (2022) 1(1):3–10. 10.1016/j.cjcpc.2021.11.002 37969558 PMC10642138

[13] Feldmann M Hagmann C de Vries L Disselhoff V Pushparajah K Logeswaran T Neuromonitoring, neuroimaging, and neurodevelopmental follow-up practices in neonatal congenital heart disease: a European survey. Pediatr Res. (2023) 93(1):168–75. 10.1038/s41390-022-02063-2 35414671 PMC9876786

[14] Smith R Roux HL Nel H Steenekamp R Brown SC Scholtz E Neurodevelopmental evaluation and referral practices in children with congenital heart disease in central South Africa. SA Heart. (2019) 16(4):324–32. 10.24170/16-4-3844

[15] Abell BR Eagleson K Auld B Bora S Justo R Parsonage W Implementing neurodevelopmental follow-up care for children with congenital heart disease: a scoping review with evidence mapping. Dev Med Child Neurol. (2023) 2:161–75. 10.1111/dmcn.15698 PMC10953404 37421232

[16] Hoskote A Ridout D Banks V Kakat S Lakhanpaul M Pagel C Neurodevelopmental status and follow-up in preschool children with heart disease in London, UK. Arch Dis Child. (2021) 106(3):263–71. 10.1136/archdischild-2019-317824 32907808

[17] Squires JE Graham ID Santos WJ Hutchinson AM, Team I. The implementation in context (ICON) framework: a meta-framework of context domains, attributes and features in healthcare. Health Res Policy Syst. (2023) 21(1):81. 10.1186/s12961-023-01028-z 37550737 PMC10408185

[18] Creech SK Hamilton EG Garza A Benzer JK Taft CT. Tailoring the implementation strategy of strength at home: an initial examination of clinician and hospital outcomes. J Aggress Maltreat Trauma. (2023) 32(7–8):1076–87. 10.1080/10926771.2023.2171826

[19] Baker R Camosso-Stefinovic J Gillies C Shaw EJ Cheater F Flottorp S Tailored interventions to overcome identified barriers to change: effects on professional practice and health care outcomes. Cochrane Database Syst Rev. (2010) 3:CD005470. 10.1002/14651858.CD005470.pub2 PMC4164371 20238340

[20] Dixit SK Sambasivan M. A review of the Australian healthcare system: a policy perspective. SAGE Open Med. (2018) 6:2050312118769211. 10.1177/2050312118769211 29686869 PMC5900819

[21] Department of Health. National strategic action plan for childhood heart disease. In: Canberra, Australia, Commonwealth of Australia (2019). p. 1–59.

[22] Hunter D McCallum J Howes D. Defining exploratory-descriptive qualitative (EDQ) research and considering its application to healthcare. J Nurs Health Care. (2019) 4(1).

[23] Damschroder LJ Reardon CM Widerquist MAO Lowery J. The updated consolidated framework for implementation research based on user feedback. Implement Sci. (2022) 17(1):75. 10.1186/s13012-022-01245-0 36309746 PMC9617234

[24] Tong A Sainsbury P Craig J. Consolidated criteria for reporting qualitative research (COREQ): a 32-item checklist for interviews and focus groups. Int J Qual Health Care. (2007) 19(6):349–57. 10.1093/intqhc/mzm042 17872937

[25] Saunders B Sim J Kingstone T Baker S Waterfield J Bartlam B Saturation in qualitative research: exploring its conceptualization and operationalization. Qual Quant. (2018) 52(4):1893–907. 10.1007/s11135-017-0574-8 29937585 PMC5993836

[26] Averill JB. Matrix analysis as a complementary analytic strategy in qualitative inquiry. Qual Health Res. (2002) 12(6):855–66. 10.1177/104973230201200611 12109729

[27] Birt L Scott S Cavers D Campbell C Walter F. Member checking: a tool to enhance trustworthiness or merely a nod to validation? Qual Health Res. (2016) 26(13):1802–11. 10.1177/1049732316654870 27340178

[28] Ballantyne M Stevens B Guttmann A Willan A Rosenbaum P. Maternal and infant predictors of attendance at neonatal follow-up programmes. Child Care Health Dev. (2014) 40(2):250–8. 10.1111/cch.12015 23294101

[29] Chorna O Baldwin HS Neumaier J Gogliotti S Powers D Mouvery A Feasibility of a team approach to complex congenital heart defect neurodevelopmental follow-up: early experience of a combined cardiology/neonatal intensive care unit follow-up program. Child Care Health Dev. (2016) 40:250. 10.1161/CIRCOUTCOMES.116.002614 PMC5285512 27220370

[30] Sapiets SJ Hastings RP Totsika V. Predictors of access to early support in families of children with suspected or diagnosed developmental disabilities in the United Kingdom. J Autism Dev Disord. (2023) 54:1–14. 10.1007/s10803-023-05996-7 PMC10159231 37142908

[31] Knutson S Kelleman MS Kochilas L. Implementation of developmental screening guidelines for children with congenital heart disease. J Pediatrics. (2016) 176(1097–6833 (Electronic)):135. 10.1016/j.jpeds.2016.05.029 27301570

[32] Alam S Ilardi D Cadiz E Kelleman M Oster ME. Impact of cardiac neurodevelopmental evaluation for children with congenital heart disease. Dev Neuropsychol. (2022) 47(1):32–41. 10.1080/87565641.2021.2009482 34894903

[33] Bolduc ME Rennick JE Gagnon I Sokol E Majnemer A Brossard-Racine M. Navigating the healthcare system with my child with CHD: parental perspectives on developmental follow-up practices. Cardiol Young. (2023) 34:1–7. 10.1017/S1047951123001051 37138527

[34] Loccoh EC Yu S Donohue J Lowery R Butcher J Pasquali SK Prevalence and risk factors associated with non-attendance in neurodevelopmental follow-up clinic among infants with CHD. Cardiol Young. (2018) 28(4):554–60. 10.1017/S1047951117002748 29357956

[35] Monteiro S Serrano F Guffey D Lopez KN De Thomas EM Voigt RG Factors affecting rates of neurodevelopmental follow-up in infants with congenital heart disease. Int J Cardiol Congenit Heart Disease. (2022) 10:100419. 10.1016/j.ijcchd.2022.100419 PMC11658356 39713596

[36] Kelsie AB Marie-Antoinette H Ailsa J Natalie O Natalie S Adam JG. Diagnostic delay in children with neurodevelopmental conditions attending a publicly funded developmental assessment service: findings from the Sydney child neurodevelopment research registry. BMJ Open. (2023) 13(2):e069500. 10.1136/bmjopen-2022-069500 36725093 PMC9896183

[37] Miller TA Sadhwani A Sanz J Sood E Ilardi D Newburger JW Variations in practice in cardiac neurodevelopmental follow-up programs. Cardiol Young. (2020) 30(11):1603–8. 10.1017/S1047951120003522 33094709

[38] Long SH Eldridge BJ Harris SR Cheung MM. Challenges in trying to implement an early intervention program for infants with congenital heart disease. Pediatr Phys Ther. (2015) 27(1):38–43. 10.1097/PEP.0000000000000101 25461764

[39] Eagleson K Campbell M McAlinden B Heussler H Pagel S Webb KL Congenital heart disease long-term improvement in functional hEalth (CHD LIFE): a partnership programme to improve the long-term functional health of children with congenital heart disease in Queensland. J Paediatr Child Health. (2020) 56(7):1003–9. 10.1111/jpc.14935 32627252

[40] Sholler GF Selbie LA Tallon M Keating J Ayer J Burchill L Australian National Standards of Care for Childhood-onset Heart Disease (CoHD Standards). 1st Edition. Heart Lung Circ. (2024) 33(2):153–96. 10.1016/j.hlc.2023.03.017 38453293

[41] Brosig C Butcher J Butler S Ilardi DL Sananes R Sanz JH Monitoring developmental risk and promoting success for children with congenital heart disease: recommendations for cardiac neurodevelopmental follow-up programs. Clin Pract Pediatr Psychol. (2014) 2(2):153. 10.1037/cpp0000058

[42] Ortinau CM Wypij D Ilardi D Rofeberg V Miller TA Donohue J Factors associated with attendance for cardiac neurodevelopmental evaluation. Pediatrics. (2023) 152(3):e2022060995. 10.1542/peds.2022-060995 37593818 PMC10530086

[43] Hennrick H Miller E Lai W Carmona V Flores A-M Olson M Effects of Implementing a Standardized Surveillance Program on Cardiac Neurodevelopmental Program Referral Outcomes [Preprint]. (2023).

[44] Michael M Scharf R Letzkus L Vergales J. Improving neurodevelopmental surveillance and follow-up in infants with congenital heart disease. Congenit Heart Dis. (2016) 11(2):183–8. 10.1111/chd.12333 26899508

[45] Roberts SD Kazazian V Ford MK Marini D Miller SP Chau V The association between parent stress, coping and mental health, and neurodevelopmental outcomes of infants with congenital heart disease. Clin Neuropsychol. (2021) 35(5):948–72. 10.1080/13854046.2021.1896037 33706666

[46] Greenhalgh T Robert G Macfarlane F Bate P Kyriakidou O. Diffusion of innovations in service organizations: systematic review and recommendations. Milbank Q. (2004) 82(4):581–629. 10.1111/j.0887-378X.2004.00325.x 15595944 PMC2690184

[47] Klein KJ Conn AB Sorra JS. Implementing computerized technology: an organizational analysis. J Appl Psychol. (2001) 86(5):811. 10.1037/0021-9010.86.5.811 11596799

[48] Komashie A Ward J Bashford T Dickerson T Kaya GK Liu Y Systems approach to health service design, delivery and improvement: a systematic review and meta-analysis. BMJ Open. (2021) 11(1):e037667. 10.1136/bmjopen-2020-037667 33468455 PMC7817809

[49] Galizzi MM Whitmarsh L. How to measure behavioral spillovers: a methodological review and checklist. Front Psychol. (2019) 10:342. 10.3389/fpsyg.2019.00342 31024368 PMC6460990

[50] DiGiacomo M Delaney P Abbott P Davidson PM Delaney J Vincent F. “Doing the hard yards”: carer and provider focus group perspectives of accessing aboriginal childhood disability services. BMC Health Serv Res. (2013) 13(1):326. 10.1186/1472-6963-13-326 23958272 PMC3765087

[51] Gallego G Dew A Lincoln M Bundy A Chedid RJ Bulkeley K Access to therapy services for people with disability in rural Australia: a carers’ perspective. Health Soc Care Community. (2017) 25(3):1000–10. 10.1111/hsc.12399 27753195

[52] Hussain R Tait K. Parental perceptions of information needs and service provision for children with developmental disabilities in rural Australia. Disabil Rehabil. (2015) 37(18):1609–16. 10.3109/09638288.2014.972586 25332090

[53] Carbone PS Behl Dd Fau—Azor V Azor V Fau—Murphy NA Murphy NA. The medical home for children with autism spectrum disorders: parent and pediatrician perspectives. J Autism Dev Disord. (2010) 40(1573–3432 (Electronic)):317. 10.1007/s10803-009-0874-5 19768528

[54] Gledhill N Scott G de Vries Nk. Routine follow-up of preterm infants in New Zealand. J Paediatr Child Health. (2018) 54(5):535–40. 10.1111/jpc.13787 29125228

[55] Sapiets SJ Totsika V Hastings RP. Factors influencing access to early intervention for families of children with developmental disabilities: a narrative review. J Appl Res Intellect Disabil. (2021) 34(3):695–711. 10.1111/jar.12852 33354863 PMC8246771

