# Supplementary material for: “It's more than just a conversation about the heart”: exploring barriers, enablers, and opportunities for improving the delivery and uptake of cardiac neurodevelopmental follow-up care
Source: Front Pediatr. 2024 May 24;12:1364190. doi: 10.3389/fped.2024.1364190 (PMC11165703; doi:10.3389/fped.2024.1364190)
Supplement: Supplementary file 1 [file Table1.pdf]

Supplementary Table 1. Template for rapid qualitative analysis of each transcript

**Summary prepared by:**		
**Interview number:**		
**Number of participants:**		
**Participant state:**		
**Participant role(s):**		
**Other relevant participant information:**		
**Question**	**Summary of key themes/response**	**Quotes/line numbers**
**PART 1**	Highlight contextualised barriers and facilitators to the implementation and delivery of neurodevelopmental support models for children with CHD throughout Australia	
What do you think are the main challenges to current service delivery?<br><br>In your opinion, what is the single largest barrier you face to being able to deliver neurodevelopmental follow-up care?<br><br>What about enablers? What works well?	*Factors which may impact the implementation and delivery of neurodevelopmental care in the <u>outer setting</u>:*<br>• *unanticipated local/national/global events*<br>• *local economic, environmental, political, and/or technological conditions for delivery of the innovation including services and infrastructure*<br>• *local social climate/customs/practices and target population – culture/beliefs, motivation, preferences, ability to access*<br>• *external partnerships/relationships, referral networks, academic affiliations, and professional organization networks*<br>• *legislation, regulations, professional group guidelines and recommendations, or accreditation standards*<br>• *funding from external entities*<br>• *external pressure/drive – societal, market, benchmarking, patient advocacy*<br>• *other*<br><br>*Attribute each factor with a + or – influence on ND follow-up care/delivery. Highlight greatest barrier with ++ if noted.*	Example quotes or references to further sections for more detail
	Factors which may impact the implementation and delivery of neurodevelopmental care in the <u>inner setting</u>:<br>• *relationships and communication within and between teams in inner setting*<br>• *positive culture – shared beliefs/values regarding meeting needs of patients, families and staff, desire to learn and share*<br>• *fit with workflows/systems*<br>• *organisation of tasks and responsibilities*<br>• *staffing and staff turnover (in hospital/clinic)*<br>• *need for change (or lack of)*<br>• *relative importance of ND care compared to other services*<br>• *incentives for implementation/service delivery*<br>• *alignment with mission/vision/goals of health service/hospital*<br>• *available resources – local funding, space/equipment*<br>• *IT infrastructure*<br>• *access to training/support to deliver care*<br>• *other*<br><br>*Attribute each factor with a + or – influence on ND follow-up care/delivery. Highlight greatest barrier with ++ if asked.*	Example quotes or references to further sections for more detail
	Factors which may impact the implementation and delivery of neurodevelopmental care related to <u>people/individuals in inner or outer setting</u>:<br>• *support (or not) of formal leaders*<br>• *support (or not) of informal/opinion leaders*	Example quotes or references to further sections for more detail

Supplementary Table 1. Template for rapid qualitative analysis of each transcript

**Summary prepared by:**		
**Interview number:**		
**Number of participants:**		
**Participant state:**		
**Participant role(s):**		
**Other relevant participant information:**		
**Question**	**Summary of key themes/response**	**Quotes/line numbers**
	• *characteristics of those who deliver ND care – capability/skills/knowledge, opportunity/availability/power, motivation*<br>• *characteristics of those who receive ND care – needs, capability/skills/knowledge, opportunity/availability/power, motivation*<br><br>*Attribute each factor with a + or – influence on ND follow-up care/delivery. Highlight greatest barrier with ++ if asked.*	
	Any other factors which may impact the implementation and delivery of neurodevelopmental care not already noted above e.g factors which bridge multiple layers, equity etc<br><br>*Attribute each factor with a + or – influence on ND follow-up care/delivery. Highlight greatest barrier with ++ if asked.*	Example quotes or references to further sections for more detail
**PART 2**	Identify gaps and opportunities in ND follow-up care	
Other pathways/models of care internationally that you think would work well for your setting?<br><br>What do you think is important for decision makers to know about how to support the delivery of this care at the system-level?<br><br>In an ideal world, what would neurodevelopmental follow-up care look like in Australia?	*Description of any gaps in current care pathways for supporting long-term neurodevelopmental needs. Establish "ideal model of care".*	Example quotes or references to further sections for more detail
**Other**	Summarise any other key discussion points here	Example quotes or references to further sections for more detail

Link to CFIR paper: https://implementationscience.biomedcentral.com/articles/10.1186/s13012-022-01245-0
