# Supplementary material for: “It's more than just a conversation about the heart”: exploring barriers, enablers, and opportunities for improving the delivery and uptake of cardiac neurodevelopmental follow-up care
Source: Front Pediatr. 2024 May 24;12:1364190. doi: 10.3389/fped.2024.1364190 (PMC11165703; doi:10.3389/fped.2024.1364190)
Supplement: Supplementary file 2 [file Table2.pdf]

Supplementary Table 2. Extract of data matrix from the barriers worksheet of Microsoft Excel

	27_BA	38_BA	08_	11_DR	12_DR	20_DR	36_BA	13_DR	14_DR	15_DR
	Queensland	Queensland	Victoria	Victoria	Victoria	Victoria	Victoria	New South Wales	New South Wales	New South Wales
Outer setting	Understanding difference between NDIS and Early Childhood Approach. ECA easier to access.	Geography and demographics, decentralisation of health services	Restrictive funding models	Lack of integration between sectors of the community	Poor integration of services across centres; Barries and silos between different primary care centres across the state	General access to ND clinics within the community is poor; not enough and too expensive; equity issue	Multiple hospitals in state providing follow-up care makes it harder to coordinate	Overall non-recognition of ND issues from CHD as a complicated problem	In Australia, Cardiology as profession doesn't take responsibility for ND care as a result of CHD or surgery for CHD – Buck passing	ND follow up care is not a national priority which impacts ability to get NHMRC grants for cardiac work
	Lack of publicly funded secondary level services e.g NGOs between GP or specialists	Inequitable service access and delivery, even in urban e.g CALD, DV, family disability	Lack of understanding about what's being done in developmental care space and lack of benchmarking in this space across states and territories	Funding questions related to sector responsibility for ND follow up leads to fragmentation and lack of coordination between services		Lack of knowledge and anxiety about medications to prescribe for cardiac kids; GPs and community paeds	Unknown role of private cardiology practices in follow-up	Early intervention relies on early assessment and improvements, particularly in community-based assessment are needed	There is too much inconsistency in ND follow up for CHD across Australian states	There is a lack of knowledge in the community, and for parents in particular, that children who have had surgery for CHD may have developmental delays later in life
		Disconnect between health and disability sectors in terms of funding models and service delivery (state vs federal); complicated cross-sector navigation for families	Lack of integration between service systems	Lack of public knowledge about CHD as a cause of ND issues		Not enough focus or funding on early childhood ND concerns generally	Not capturing outcomes of ND follow-up care (to understand what works best)	Funding levels mean that it is impossible for one service to have ownership/manage all aspects of patient care	There is not enough funding for ND follow up in Australia	Unbalanced/inequitable priorities in funding – surgery vs follow up care
		Health services not good at understanding what is required to access disability supports; often different to diagnosis letter – needs to be individualised based on function and support needed; need to build partnerships for greater understanding of eligibly and services available	Victoria health system is very 'hospital focused', particularly ED; community services not well funded and are a 'patchwork unstandardised model'	Different layers of funding with little integration		Lack of support for regional community hubs	Lack of funding for non-acute care	Issues in providing services in rural and regional communities and with priority populations; Related to regional/rural and priority populations is that there is need to invest more heavily in coordination	Some hospitals focus on assessment without much thought about access to services as a result of the assessment	Imbalances/inequities in funding for follow up care for similar high risk cohorts – pre-term vs CHD
Inner setting	Lack of reminders for follow-up on pathway	As is not a structured, systematic process kids can fall through gaps esp those not high risk or regional/remote	Disjointed communication/cooperation within hospital cardiac and developmental services	Size of cohort makes it difficult/expensive for there to be a single consolidated comprehensive ND follow up program	Lack of funding is the key challenge to deploying a comprehensive model of care for CHD, rather than opposition from clinicians or cultural challenges	Lack of co-ordination/communications between cardiologists and paediatricians	Lack of funding/access to psych screening and support	ND issues and assessment is intertwined with Mental health and psychological issues, assessment and treatment	Perception by hospital executive that psychology and neurodevelopmental care for cardiac is good optional extra rather than essential	Not funded for follow up, so need to engaged in fundraising activities
	Complexity of pathway	Limitations in resources/staff	Reliance on 'home team' (e.g., cardiology) to investigate/recognise/pass on indicators ND and refer in	Successful ND program needed dedicated and specifically employed/funded staff to make it happen	Lack of allied health clinicians/predominantly doctors and nurses in Cardiology department means that focus is on medical outcomes and ND not really considered	Low staffing capacity means long waitlists – Cardiology service knows to refer to community options first	Lack of specialist CHD knowledge psych providers	Bespoke pathway for each individual patient but may not be consistent/standardised	Despite the demonstrable impact of the service in terms of research publications and research funding, the value of providing neurodevelopmental care for cardiac is not well understood by hospital service executives with limited resources	School aged children receive regular medical follow up by cardiologists but this does not generally extend to developmental follow up – it's not the cardiologists role
		Lack of nursing resourcing	Long waitlists	Lack of buy in to fund large scale intervention programs for CHD Neurodevelopmental follow up		Development and implementation of ND care across the hospital has been ad hoc and reliant on staff advocacy; this is inequitable	Lack of AH therapists to deliver care	unclear about knowledge that GPs or general Paeds have of pathways/treatments for ND	Pathways do not extend beyond young childhood	Issues accessing to space and equipment to do ND follow up effectively; e.g., room availability, access to MRI
