# Supplementary material for: “It's more than just a conversation about the heart”: exploring barriers, enablers, and opportunities for improving the delivery and uptake of cardiac neurodevelopmental follow-up care
Source: Front Pediatr. 2024 May 24;12:1364190. doi: 10.3389/fped.2024.1364190 (PMC11165703; doi:10.3389/fped.2024.1364190)
Supplement: Supplementary file 3 [file Table3.pdf]

Supplementary Table 3. Table mapping themes to CFIR constructs and domains. Most barriers were observed in the outer setting; most enablers were in the individual domain.

Theme	CFIR Construct	Outer setting	Inner setting	Individuals	Innovation	Process
Fragmentation of care	Partnerships and connections; relational connections; communications					
Low priority of developmental care	Relative priority; external pressure; innovation deliverers (motivation)					
Blurred lines of responsibility	Policies; work infrastructure; relative priority; innovation deliverers (motivation)					
Lack of standardisation in process and guidance	Policies; performance-measurement pressure; reflecting & evaluating					
Accessibility and availability of developmental services	Local conditions; available resources; structural characteristics					
Workforce and staffing	Work infrastructure; available resources;					
Environmental and socioeconomic context	Local conditions					
Sociocultural characteristics of families	Innovation recipients (capability, opportunity and motivation)					
Family centred models of care	Recipient-centredness; innovation recipients (need); innovation adaptability; engaging; innovation complexity					
Funding and resources	Financing; available resources					
Publicly funded insurance scheme	Financing; Policies					
Knowledge about developmental care	Innovation deliverers (capability); innovation recipients (capability)					
Passionate and generous healthcare providers	Innovation deliverers (motivation)					
Improving systems and coordination	Adapting					
Leadership and champions	Individuals (leaders)					
Leveraging existing programs and pathways	Adapting, available resources; relational connections					
Relationships, networks and interprofessional collaboration	Relational connections; communications; recipient-centredness					
Skilled multidisciplinary teams	Work infrastructure; teaming; innovation deliverers (capability); access to knowledge and information					
