# Supplementary material for: “It's more than just a conversation about the heart”: exploring barriers, enablers, and opportunities for improving the delivery and uptake of cardiac neurodevelopmental follow-up care
Source: Front Pediatr. 2024 May 24;12:1364190. doi: 10.3389/fped.2024.1364190 (PMC11165703; doi:10.3389/fped.2024.1364190)
Supplement: Supplementary file 4 [file Table4.pdf]

Supplementary Table 4. Table of additional quotes to support barriers and enablers identified in the interviews

Barrier or enabler	Ref	Explanatory Quotes	Interview participant
**Funding and resources**	A1	*"I think funding's the major limiting factor and our biggest barrier behind actually extending our neurodevelopmental follow-up. And it's been really hard overcoming that challenge."*	P41, Neonatologist, Western Australia
	A2	*"I feel like the lack of resourcing creates this really high-level of risk for everybody. For families, and for the clinicians who are just like mice on wheels, powering through the assessments."*	P37, Child Development Unit Lead, South Australia
	A3	*"Our problem is that we're not funded for follow-up. So, we raise funds ah, from donated funds. We have an annual fundraising gala. And we have another cycling event, and we just ask families for money."*	P15, Medical Director, New South Wales
**Workforce and staffing**	B1	*"It is hard to attract and retain good experienced staff and obviously kids with congenital heart disease is an area of specialist practice."*	P32, Speech Pathologist, Northern Territory
**Prioritization of long-term developmental care**	C1	*"Not always, but at times, has it been difficult at a hospital leadership level, like a hospital executive level to advocate for additional FTE [staffing] that is desperately needed? Absolutely. Absolutely."*	P14, Psychologist, New South Wales
	C2	*"Any time that I've tried to leverage business cases … the developmental paediatrics just seems so far from being a priority."*	P1, Allied Health Lead, Queensland
	C3	*"It's not really valued. The hospital, the health system. … The fact it's seen as, optional, that's the barrier. It needs to stop being a nice thing to do but seen as essential."*	P15, Occupational Therapist, New South Wales
	C4	*"In one of our [community] sites, there's been a nurse who didn't really see herself as a paediatric development nurse, but she's run with it and she's a great clinician, a great support to those families and she is excellent, when it was never you know, her thing."*	P24, Psychologist, Queensland
**Improving hospital systems and coordination**	D1	*"So, this has helped quite a lot in getting our families to engage…we have removed that barrier and they can just come directly to us."*	P30, Paediatric Nurse, Northern Territory
**Relationships, networks and interprofessional collaboration**	E1	*"Our team of paediatricians are beautiful, like all of them, I cannot fault one. They're really responsive to us, and they actually do respect and listen to us."*	P30, Cardiac Nurse, Northern Territory
**Passionate and generous health care providers**	F1	*"We have people who have been involved in the work for a very long time and despite all the challenges are still sitting at the table, and giving it everything they've got which is, you know a real testament to them."*	P3, Clinical Nurse Consultant, Queensland

**Knowledge and understanding of neurodevelopmental care (providers)**	G1	*"I don't think we're good at that. I don't think we're actually good at saying, 'This is what we normally expect for cardiac babies and we are going to walk with you and watch out for those things and do something about it early with you.'"*	P6, General Paediatrician Queensland
**Blurred lines of responsibility**	H1	*"In the States, the heart institutes and the heart centres say, 'this is our responsibility this is part of cardiac care'; I do not see that happening systematically in Australia."*	P14, Psychologist, New South Wales
	H2	Participant recounting a mother's challenge in seeking care: *"The cardiologists have said he's fine. Paediatricians aren't interested but he's got a lot of academic challenges. And she didn't know how to get help. And that really stuck with me."*	P11, Neonatologist, Victoria
**Knowledge and understanding of neurodevelopmental care (family)**	I1	One participant recalled the struggles of a mother in dealing with her child's issues at school: *"And she called me to say, 'I remember you did a study on my child. And nobody believes us, that his heart might have played a part in why he's struggling now.'"*	P11, Neonatologist, Victoria
	I2	*"You don't just throw a kid on a bike, you usually have training wheels and you run beside them, and I don't think there are training wheels, and I don't think anyone's running beside the vast majority of families."*	P38, Paediatric Disability Lead, Queensland
	I3	*"Some mothers are really great advocates for their children. And they've got great understanding as to what's going on with their child, … and I think when you've got a very vocal, well-spoken parent who has got a good understanding, that actually helps children get more services. Or they make sure that they're getting what they need."*	P10, Occupational Therapist, Queensland
**Lack of standardised processes and guidance for developmental follow-up care**	J1	*And I think we probably couldn't say we have a model of care. I don't think we do. I think these kids come – they're like – we deal with it case by case, which is not the best.*	P29, Physiotherapist, Northern Territory
	J2	*"I've always been worried about when we discharge them, just making sure families stay on that screening pathway because we have no line of sight to that."*	P1, Allied Health Lead, Queensland
**Leveraging existing programs and pathways to improve access**	K1	*"I think that there's actually lots of vulnerabilities in the community that have this exact same challenge, which makes me think that really what would be most useful is a more integrated secondary level approach, to be honest."*	P27, Child Development Unit Lead, South Australia
