# Supplementary material for: “It's more than just a conversation about the heart”: exploring barriers, enablers, and opportunities for improving the delivery and uptake of cardiac neurodevelopmental follow-up care
Source: Front Pediatr. 2024 May 24;12:1364190. doi: 10.3389/fped.2024.1364190 (PMC11165703; doi:10.3389/fped.2024.1364190)
Supplement: Supplementary file 5 [file Table5.pdf]

Supplementary Table 5. Quotes to support each strategy for improving follow-up care

Opportunity: Use evidence to advocate for need and value of investment

Strategy	Explanatory Quotes	Interview participant
**Emphasise long-term return on investment for early intervention and developmental care**	*"There's so much literature now about neurodevelopmental outcomes, and how patients with congenital heart disease are affected, the first thing is to highlight the problem. And so that's – and it's a problem that we can do something about, if we were resourced and we had the right amount of resources and people to tackle it."*	P35
	"There's a big capacity to provide, on a dollar for dollar, bang for buck kind of area, a lot more [return] in paediatric services [compared to adult services]."	P19
	"I want them to know that if they invested early in these kids, they would come back and pay them taxes for the rest of their life. They will get their return, yeah, absolutely. If you can support kids to grow and develop in healthy environments, they will grow up, they will get qualifications, they will work, they will pay taxes, they will contribute to society."	P11
	"Evidence says that investing in getting it right at the start makes a tremendous difference both from a health perspective but from social systems, education, um, youth justice; the whole thing."	P1
	"I think the boss needs to know that it's important in general, that the evidence is there, that this should be part of standard care. And that the boss should know that it's about advocating for the resources to give the right care at the right time, at the right place."	P21
**Develop standardised guidance and outcome measures**	*"So, I think a framework sort of trying to detail what to do, when to do it, how services can be accessed, and also a method of – a plan to implement that, and to fund that kind of framework. … something that's applicable in each state, which may not be exactly the same – I'm sure won't be exactly the same across each state… having something established and structured would make a big difference."*	P35
	"Ideally moving to some sort of national guideline around follow-up for this group, that the dates or the follow-up should align, because it would be useful to be able to pull research information outcomes. And have each service following up at the same time points. Ideally with the same set of assessments."	P15
	"Some kind of framework that everyone agreed upon with, you know, relevant stakeholders involved in designing it, sort of an evidence based or a consensus statement about what should be provided optimally and what as a minimum that should be provided and some guidance for how to achieve that."	P16

Supplementary Table 6. Quotes to support each strategy for improving follow-up care

Opportunity: Build partnerships to break down silos

Strategy	**Explanatory Quotes**	**Interview participant**
**More consistent approach to care nationally**	*"I think a greater consistency and coherence across Australia. I don't think there's one model that will fit across Australia and I deeply respect different states doing things differently, but there can be some universal aspects to it, you know? Universal communication around the importance of it, universal communication on what a developmental assessment is and means and can help with."*	P14
	"Much more of comprehensive approach, both through and post-diagnosis, and then pathways that families are supported to navigate that are really clear to them, they know what the next step is, what the next step is and someone is there to support them to work through those different pathways as relevant."	P37

Opportunity: Increase funding and resources

Strategy	**Explanatory Quotes**	**Interview participant**
**Increase access to secure funding**	*"I think, if you get that sort of secure funding and you get good enough people, they'll make it. They'll make the service work for their particular population."*	P26
	"It's about having the resources you need to really sit with families in that space and ensure that they feel empowered to support their child and to advocate for their child's needs."	P2
	"…having FTE [staffing] dedicated to this care that is part of the operational budget of the hospital."	P14

Opportunity: Grow capacity of specialist workforce

Strategy	**Explanatory Quotes**	**Interview participant**
**Provide training to community providers**	*"Like some educational package for everyone in the community to know how to screen these patients appropriately so that they can then refer them when it's required or actually then resources on how to manage it otherwise."*	P44
	"Well, I think you can't put a neurodevelopmental specialist on every corner. But you can certainly improve local Allied Health services. And you can certainly improve outreach services."	P39

Supplementary Table 6. Quotes to support each strategy for improving follow-up care

Opportunity: Adapt care provided to meet family needs

Strategy	**Explanatory Quotes**	**Interview participant**
**Provide education, resources and coaching to families**	*"Because if you are an empowered and competent parent caring for a vulnerable child, there's going to be much better mental health outcomes for your family."*	P6
	"Parental education about normal milestones and about how to facilitate your child's development, but also how to identify early when they're starting to lag a little bit, and then how to access those supports."	P32
	"I think having a structured resource, you know, I think something that is online that parents themselves can access that you can point them towards that it is credible and it's backed up."	P13
	"I'd love to see education to families so that they can feel empowered to advocate for what the children need."	P11
	"And if we can offer tools for education, tools for, ah, assessment, that can be universally accessed and that is language that parents of all levels of education can access, – in my view will be the principal way to get people to that ideal neurodevelopment."	P13
**Develop and support new models of care**	*"An obvious opportunity for change there is virtual. And how do we support, you know, different workforce models that allow a mix of face to face, um, and support via virtual means."*	P17
	"I love the idea of the one stop shop. You know, so if we couldn't change resourcing, co-locating all services together so families with concerns can turn up	P2
	"Have some integrated paediatric hub locally that focuses on development broadly ... co-located child health nurses, child development service, paediatricians with enough rooms for us to run enough clinics that we actually can get through our wait lists and see everybody in an adequate time."	P44
	"It would be a wrap-around kind of service that not only they get screened, assessed, diagnosed, but then they can also receive treatments, probably in the same house or the home – same service rather, than the fractured system that is like a jigsaw puzzle that often doesn't connect."	P31
**Expand developmental services**	*"So, I think going into the early school years is vital. So, you know, reviewing them at five and then perhaps at eight as well, because there are different problems that you find when you go looking for them at those ages that we just can't detect at three."*	P19
**Implement care navigation and coordination**	*"Invest in coordination. You know, whether it's a nurse coordinator or administrative coordinator, that there is a point person who is interested in the cardiac group of patients, for kids, and can coordinate what's going on and create connections between the major centres or the paediatricians or the local hospital or the schools, and so on."*	P13
**Co-design with families**	*"We would have an individualised developmental follow-up plan or care plan that would take into account the families' needs."*	P16

Supplementary Table 6. Quotes to support each strategy for improving follow-up care

Opportunity 6: Better integrate data and leverage existing systems

Strategy	**Explanatory Quotes**	**Interview participant**
**Improve and connect data and communication**	*"So having something that is actually nationwide is the way to go and having a whole record of actually what's happening with those children so that they're not actually lost to follow-ups. I mean, a lot of these children need good monitoring."*	P10
	"I feel like a much more robust health record communication system, would be really terrific. Um, and – and one that families could access too."	P3
	"I think for data sharing and research more collaboration, and the things that tend to stop at the state border could be improved upon I think.	P16
	"I think if you could build better networks and ways of communicating with each other that are easier than snail mail and letters it would make things a lot easier."	P44
**Use existing pathways**	*"I think trying to make it as streamlined as the neonatal program is, where we don't have to think about it every time for every patient, it just happens, that's how to capture everybody"*	P14
