# Supplementary material for: “It's more than just a conversation about the heart”: exploring barriers, enablers, and opportunities for improving the delivery and uptake of cardiac neurodevelopmental follow-up care
Source: Front Pediatr. 2024 May 24;12:1364190. doi: 10.3389/fped.2024.1364190 (PMC11165703; doi:10.3389/fped.2024.1364190)
Supplement: Supplementary file 6 [file Datasheet1.pdf]

Supplementary Material 1. CHD LIFE+ Semi-Structured Interview Guide

Aims:

- Highlight contextualised barriers and facilitators to the implementation and delivery of neurodevelopmental support models for children with CHD throughout Australia
- Identify gaps in service provision and support that may benefit from new models of care or implementation strategies

Structure:
The interviews will be semi-structured, with open-ended questions which are adaptable to reflect the responses being provided. The conversation should explore issues as they are raised, with guidance and prompting as required. Interviewers/facilitators will ensure discussion progresses in a timely, yet informative manner. Interviews will last no more than 60 minutes, focus groups will last a maximum of 90 minutes.

Content:
Review aims of session, participant information sheet, particularly confidentiality, consent, privacy and right not to participate. Begin recording and ask for verbal consent (virtual/phone) or check and collect signed consent forms (in person). A guide for the content and design of questions is provided in Table 1. All key questions in Part 1 should be covered. Part 2 may not need to be covered in each interview, depending on participant answers to questions in the previous section. The content of the group and individual interviews will be based on (a) the models of care and characteristics found during evidence mapping review and (b) the Consolidated Framework for Implementation Research[1].

References
Damschroder LJ, Aron DC, Keith RE, Kirsh SR, Alexander JA, Lowery JC. Fostering implementation of health services research findings into practice: a consolidated framework for advancing implementation science. Implementation science. 2009 Dec;4(1):1-5.

Section 1. Highlight contextualised barriers and facilitators to the implementation and delivery of neurodevelopmental support models for children with CHD throughout Australia

Great, so I just wanted to start by learning a bit about you and your experience with congenital heart disease and follow-up care.

- Could tell me a bit about your current role and how it's related to supporting children with congenital heart disease? *(Probes: How long?...How get involved?)*

So, now we're going to think about the challenges, but also the enablers to delivering neurodevelopmental follow-up care. Firstly, I want you to think just within the **scope of your own practice/program and hospital….**

What do you think are the main challenges to current service delivery?

- Infrastructure – physical, work, IT
- Networks and communications
- Leadership commitment, champions
- Culture – patient-centredness, learning, tension for change, compatible and priority
- Goals/feedback and rewards
- Available resources – funding, staff, time, space, knowledge
- Model itself e.g. flexibility, quality

(Probes: How does this impact…? What do you mean by…? Can you tell me how…? Can you give me an example of…?)

What might be some suggestions for how to overcome these challenges?

Okay, so what about enablers? What works well?

So now I want you to think more **broadly about neurodevelopment as a whole, considering** its place in the health system in the State and Australia.

What do you think are the main challenges to current service delivery?

- Policies/incentives – QI, benchmarks, guidelines, associations, insurance
- Networks/partnerships
- Recipients – advocacy, needs, prefs
- Social-cultural, socio-geographic, socio-economic
- Market forces -supply/demand, competition

(Probes: How does this impact…? What do you mean by…? Can you tell me how…? Can you give me an example of…?)

Okay, so what about enablers? What works well?

Section 2: Gaps in care

Okay I just wanted to finish up with some big picture questions trying to think about the future

- In your opinion, what is the single largest barrier you face to being able to deliver neurodevelopmental follow-up care?

- What do you think is important for decision makers to know/understand about how to support the delivery of this care at the system-level? (*Where are our best opportunities?)*

- In an ideal world, what would neurodevelopmental follow-up care look like in Australia?

Do you have any questions for me?

Thank you for participating in the interview today. Please feel free to get in contact with the research team if you have any questions. We're also planning to hold a national workshop in a few months' time. Would you be interested in hearing about that?

[end of interview]
